# Sequestration of Pb(II) Ions from Aqueous Systems with Novel Green Bacterial Cellulose Graphene Oxide Composite

**DOI:** 10.3390/ma12020218

**Published:** 2019-01-10

**Authors:** Alfred Mensah, Pengfei Lv, Christopher Narh, Jieyu Huang, Di Wang, Qufu Wei

**Affiliations:** Key Laboratory of Eco-textiles, Ministry of Education, College of Textiles and Clothing, Jiangnan University, Wuxi 214122, China; alphredmax@gmail.com (A.M.); 18861800875@163.com (P.L.); doll6000000@yahoo.com (C.N.); 18861823219@163.com (J.H.); wd544187592@163.com (D.W.)

**Keywords:** bacterial cellulose, graphene oxide, esterification, trap entanglement, electrostatic interactions, adsorption, desorption

## Abstract

In this study, a novel green adsorbent material prepared by the esterification of bacterial cellulose (BC) and graphene oxide (GO), richly containing hydroxyl, alkyl, and carboxylate groups was characterised by FTIR (Fourier Transform infrared spectroscopy), XRD (X-ray diffraction), SEM (Scanning electron microscopy) and TGA (Thermo-graphimetric analysis). The specific surface area (SSA) and pore size distribution (PSD) analysis of materials were also analysed. Batch experiments–adsorption studies confirmed the material to have a very high Pb^2+^ removal efficiency of over 90% at pH 6–8. Kinetic studies showed that the uptake of metal ions was rapid with equilibrium attained after 30 min and fitted well with the pseudo-second-order rate model (PSO). Isotherm results with a maximum adsorption capacity (Q_max_) of 303.03 mg/g were well described by Langmuir’s model compared to Freundlich. Desorption and re-adsorption experiments realised that both adsorbent and adsorbates could be over 90–95% efficiently recovered and reused using 0.1 M HNO_3_ and 0.1 M HCl.

## 1. Introduction

Heavy metal ions pollution from industries (sectors such as metallurgy, pharmaceutical, chemical industry, and petroleum refining etc.) and individual domestic activities present a grave threat to humans and aquatic and other life forms. Metal ions such as lead (Pb^2+^), zinc (Zn^2+^), cadmium (Cd^2+^), manganese (Mn^2+^), silver (Ag^+^) and mercury (Hg^2+^) have been enlisted by the Environmental Protection Agency (EPA) as priority pollutants [1]. These ions are deposited into the atmosphere, the earth (soil) and water bodies in large amounts by industries, but also, some of these metals exist naturally in water bodies in minimal quantities. At minimal levels, metals like Zn^2+^, Cu^2+^, Fe^2+^ and Fe^3+^ as well as a few others are good for very essential biological processes in human physiology [2]. However, Pb^2+^, particularly, at higher concentrations found in wastewater streams could cause numerous diseases including anaemia and nervous system degeneration [3].

The concerns of heavy metal pollution have in recent times led to many varied technology introductions to help remediate the issues. These technologies include reverse osmosis, ultrafiltration, ion exchange, coagulation, floatation, chemical precipitation, electrodialysis, flocculation, evaporative recovery, sorption (absorption and adsorption) technologies and many others [4,5,6,7]. Nevertheless, challenges such as complicated protocols and the release of toxic wastes during synthesis have called for a more efficient, less harmful processes with specific focus on the mass transport of contaminants in the fastest way possible; exhibiting effective selectivity and capacity for specific contaminants; be of low-cost and modest to engineer; and minimisation and maintenance of operational requirements [8].

Adsorption is essentially toxic-free, low cost, simple to operate, flexible and conducts rapid adsorption [9]. Many research reports have revealed new adsorbents of heavy metal ions including inorganic materials, bio sorbents and activated carbon [10,11,12]. The most effective way of getting rid of heavy metal ions from aqueous systems is adsorption and this is crucial since metal ions cannot be degraded by chemical reactions and bioprocess [13].

Sorption refers to a metal ions’ phenomenon in which they associate (ranging from electrostatic to covalent) with one that is peripherally available or more functional groups on the sorbent material [2].

Bacterial cellulose (BC), as a biosynthesised polymer, has peculiar properties which are abundantly reported in the literature and that have led to a soaring interest in their technical applications besides the already known medical functions [5,6,14,15,16,17,18,19,20,21,22,23,24]. BC has the capacity to act as a host biopolymer to receive other various guest molecules or inorganic particles to be incorporated into its matrix [15,17,25,26]. For this reason, many BC hybrid composites are now studied for different applications such as photo-catalytic nano-materials, optically transparent films, magnetic materials and adsorbent materials, especially for heavy metals and many others.

Because BC is a natural polysaccharide like plant cellulose, chitosan and others, which have successfully been used as materials for adsorption, it stands a greater chance to be better used to prepare a new composite adsorbent for metal ions. Further modification to introduce functional groups such as amine/amides, carboxyl and thiol groups will offer excellent remediating capacities through their hydroxyl group sites and also owing to the characteristic nano-reticular three-dimensional structure of BC [27]. Established modes of modifications of BC include grafting, ethylation, amination and densification [28]. 

Graphene oxide (GO) has abundant oxygen-containing functional groups such as hydroxyl, carbonyl, carboxyl and epoxide on the surface, enabling them to form strong complexes with metal ions. These characteristics allow GO to act as an adsorbent for heavy metal ions, organic solvents, dyes and oils removal and pre-concentration [7,29,30,31,32,33,34]. Several reported graphene composite publications are in the areas of electronic and catalytic usages and water purification via adsorption [10,35,36,37,38,39,40]. Part of the soaring interest is due to the discovery of its exceptional physicochemical characteristics as a result of its significant structure; being a lightweight material which can withstand high pressure [10,30], has a higher specific surface area and low production cost [41]. It is well known that electrostatic interaction is the mechanism behind the cationic heavy metal remediation; between metal cations and negative surface charge and/or electrons of the composite [10]. 

Few reports are available on bacterial cellulose–graphene oxide composites as an adsorbent in water pollution remediation in spite of the overwhelming publications on the application of graphene oxide (GO) and graphene nanosheets (GN) composites in numerous diverse fields. 

Wang et al. reported a superabsorbent novel BCGO (Bacterial cellulose Graphene oxide) aerogel which exhibited an excellent property for the absorption of organic liquids [16], which offers a good background for the exploration of BCGO’s adsorptive capabilities. 

The purpose of this present work was to synthesise a novel superadsorbent via an innovative and improvised green approach for the removal of Pb^2+^ metal ions contaminants in aqueous solution. Adsorbent before and after the adsorption process was characterised using FTIR, SEM, XRD and TGA analysis. The adsorption conditions for the adsorbent were optimised by varying several experimental parameters such as pH, contact time, initial concentration and temperature by a batch adsorption method. 

## 2. Materials and Experiments

### 2.1. Chemicals and Reagents

Chemicals for the synthesis of BC were derived from Jiangnan University (in-house laboratory), Wuxi, China. BC; saccharose, peptone, citric acid, Na_2_HPO_4_, KH_2_PO_4_ and MgSO_4_ were all purchased from Sinopharm Chemical Reagents Co., Ltd., Beijing, China. The bacterial strain was Gluconacetobacter xylinus 1.1812.

For GO, reagents such as graphite, sodium nitrate (NaNO_3_), potassium permanganate (KMnO_4_), sulphuric acid (H_2_SO_4_), hydrogen peroxide (H_2_O_2_) and hydrochloric acid (HCl) have been the long-standing chemicals for the preparation of GO by the ever-present Hummers method and its numerous modifications; however, we came across a green and completely efficient approach which substitutes NaNO_3_, KMnO_4_, H_2_O_2_ and HCl for the strong oxidant K_2_FeO_4_ reported by [42]. Reagents were used as procured without purifications from Sinopharm Chemical Reagent Co., Ltd., Beijing, China.

### 2.2. Synthesis of Pure BC

Pure BC culture (static condition) medium consisted of 68.2% (*w*/*v*) saccharose, 21.8% (*w*/*v*) peptone, 2.71% (*w*/*v*) citric acid, 2.71% (*w*/*v*) Na_2_HPO_4_, 4.1% (*w*/*v*) KH_2_PO_4_, 0.5% (*w*/*v*) MgSO_4_ and adjusted to a pH of 3.5 with hydrochloric acid. The culture medium was autoclaved at 130 °C and then allowed to reach room temperature. Ethanol was added after sterilisation to improve the growth of the cellulose gel. The strain Gluconacetobacter xylinus was inoculated and cultivated at 30 °C for 20 days at room temperature.

### 2.3. Synthesis of GO

K_2_FeO_4_ (3.6 g) was introduced into the reactor flask together with 82 mL of H_2_SO_4_ at room temperature. Graphite (0.68 g) was later added to the mixture and magnetically stirred for 1 h, still at room temperature. The solution underwent centrifugation using deionized water after recycling of the sulphuric acid. This was repeated four times at 8000 rpm for 5 min to ensure the pH of the supernatant was close to 7. The washed GO suspension was sent for freezing in a refrigerator at −30 °C in a polytetrafluoroethylene container. It was then further dried directly for 24 h with a vacuum drying machine.

### 2.4. Synthesis of BC/GO Membranes

**Figure 1:**
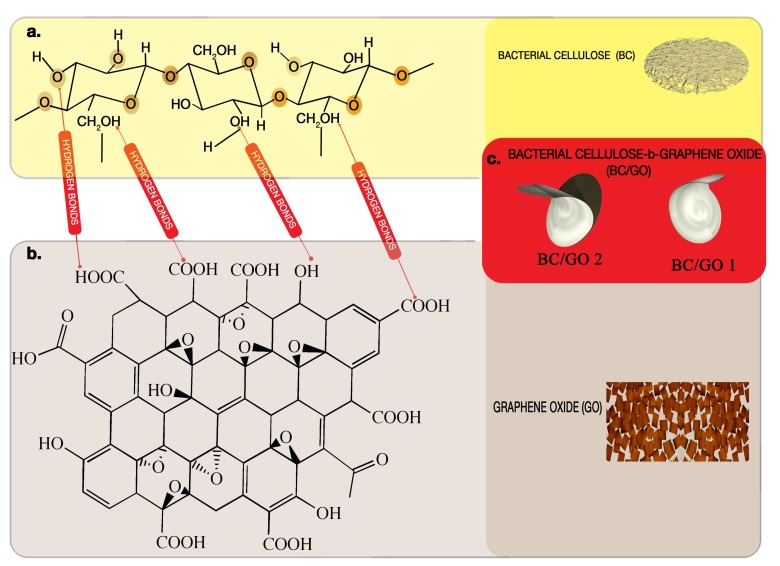
Synthesis of BC/GO membranes and their chemical structures. (**a**) Bacterial Cellulose (BC), (**b**) structure of Graphene oxide (GO) and (**c**) BC/GO (composites 1 and 2), obtained after hydrogen bonding and entanglement.

BC/GO composite membranes were prepared by two main approaches. BC/GO 1 was synthesised by facile one-pot in-situ biosynthesis, while BC/GO 2 was synthesised by what is newly termed in this paper as a “trap-entangling” approach, all under static conditions. Structure of materials are displayed in Figure 1.

#### 2.4.1. BC/GO 1 (Plain)

The culture medium for this biosynthetic membrane was produced from 50 g/L saccharose, 16 g/L peptone, 2 g/L citric acid, 2 g/L NaHPO_4_, 3 g/L KH_2_PO_4_ and 0.3 g/L MgSO_4_. The solution was autoclaved at 130 °C for 1 h to sterilise the medium.

After this, 0.1 g of GO was deposited into the culture solution and magnetically stirred for 1 h before sonication for a further 2 h to ensure absolute dispersion; at this point 10 mL of the seed broth (Gluconacetobacter xylinus) BC was inoculated into a 500 mL Erlenmeyer flask containing 100 mL of GO dispersed medium. These flasks were then incubated under static conditions at 29–30 °C for 20 days.

**Scheme 1:**
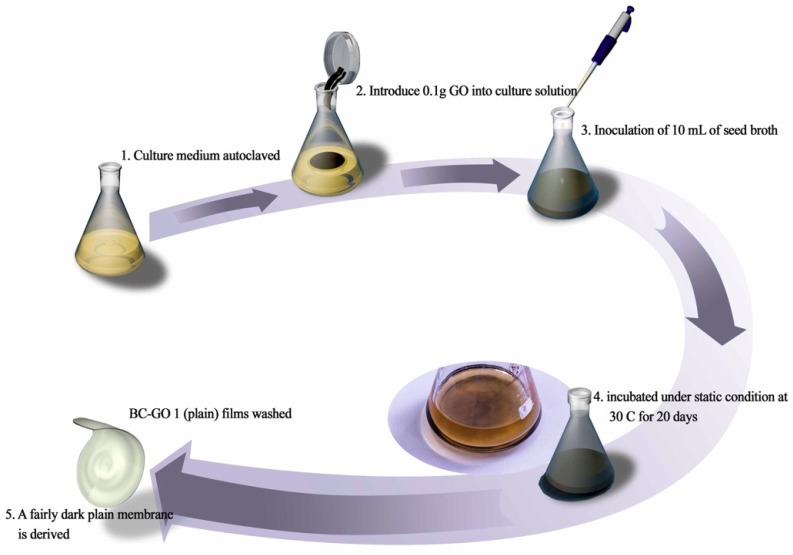
Synthesis of BC/GO 1.

The schematic diagram of the synthesis process of BC/GO 1 membrane is illustrated in Scheme 1.

The harvested membrane was washed with deionized water after 20 days and boiled in a 0.5 M NaOH solution at 80 °C for 60 min to eliminate impurities such as medium components and residual cells. The final membrane appeared with a plain alabaster white complexion.

#### 2.4.2. BC/GO 2

The same quantities and conditions for the synthesis of BC/GO 1 were used to produce BC/GO 2; however, the approach was methodically improvised. With the solution autoclaved and the 0.1 g GO dispersed, it was magnetically stirred and sonicated for 2 h for complete dissolution. The solution was transferred into a petri dish in a strictly non-contaminative atmosphere and then sterilised again by autoclaving under static conditions before the seed broth was inoculated and incubated under static conditions at 29–30 °C for 20 days. This approach derives the name “trap-entanglement” from the fact that under static conditions, the GO particles tended to precipitate (after many days) to the bottom of the culture solution in the petri dish and were fetched by the process of bacterial growth to form a bonded layer with the BC. With the petri dish offering a broader surface stretch of the culture medium, the bacteria accumulated mats of cellulose on the surface of the nutrient broth which is an oxygen-rich air–liquid interface while the extruded sub fibrils of cellulose trap the GO particles beneath as they crystalised. The GO nanoplates are evenly distributed and bound by the BC nano-fibril growth in a spiderweb-like manner [43].

**Scheme 2:**
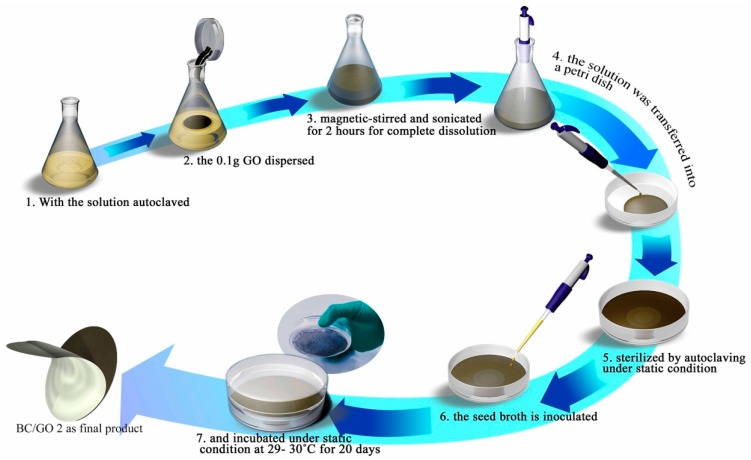
Synthesis of BC/GO 2.

The graphical illustration of the procedure to synthesise BC/GO 2 (black) is shown in Scheme 2.

### 2.5. Characterisation

Concentrations of the heavy metal ion solutions before and after equilibrium were determined using an Atomic Absorption Spectrometer TAS-990 (Beijing Purkinje General Instrument Co., Ltd., Beijing, China). For pH, solutions were checked using a Hanna pH meter (Goldpoint Instrument Company Ltd., Shanghai, China) with a glass electrode.

#### 2.5.1. Fourier Transform Infrared (FTIR) Spectroscopy

A total of 5.0 mg of each of the material samples BC, GO, BC/GO 1 and BC/GO 2 were examined. FTIR spectral analysis of this experiment employed a Nicolet MAGNA-IR Spectrophotometer (Thermo Electron Corporation, Waltham, MA, USA) in the range of 400–4000 cm^−1^ with 32 scans per sample.

#### 2.5.2. Scanning Electron Microscopy (SEM)

SEM images of sample surfaces were analysed using a SEM-SU1510 (Hitachi Company, Tokyo, Japan) scanning electron microscope operating at a tungsten filament voltage from 15 to 20 keV with a secondary electron (SE) detector. Before examination, the samples were dried in an oven at 90 °C for 1 h and later brought to cool in a desiccator. Then, samples were fixed on sample holders containing a graphite ribbon and subsequently sputter-coated with gold in a modular high-vacuum coating.

#### 2.5.3. Thermogravimetric Analysis (TGA)

Materials thermogravimetric analysis (TGA) were recorded using an analyser from Mettler Toledo Co., (Shanghai, China) in static air at a heating rate of 10 °C/min from room temperature to 800 °C. 

#### 2.5.4. X-ray Diffraction (XRD)

The crystalline structure of GO, BC, BC/GO 1 and BC/GO 2 nanocomposites were characterised by XRD. The diffractograms were obtained by a LabX XRD-6100 X-ray diffractometer (Shimadzu, Kyoto, Japan) equipped with CuKα radiation; operating voltage of 40 kV and a current of 30 mA. The diffractograms were obtained with a 2Ø (Bragg angle) range from 4 to 70° at a scan rate of 4°·min^−1^.

#### 2.5.5. Specific Surface Area and Pore Size Distribution

To determine the specific surface area of the adsorbent materials, measurements were performed on a surface area and pore analyser Micromeritics TriStar II 3020 (Micromeritics Instr. Corps., GA, USA) using N_2_ adsorption–desorption isotherms at 77.35 K. Samples of BC, BC/GO 1 and BC/GO 2 were degassed at 30 °C for 24 h under vacuum at 0.016 mmHg prior to measurement. The N_2_ amount adsorbed and desorbed onto samples were measured over a wide range of relative pressures (P/P_0_ = 2 × 10^−5^–1.0), whereby P represents the equilibrium pressure and P_0_ is the saturation pressure. The specific surface area was determined and calculated using the Brunauer, Emmett and Teller (BET) method and equation [44], while pore size distributions were evaluated according to the Barrett, Joyner and Halenda (BJH) method [45].

### 2.6. Adsorption Experimental Studies

The adsorption behaviour of the prepared composite materials BC, BC/GO 1 and BC/GO 2 for Pb^2+^ metal ions (mono-component systems) were evaluated on experimental parameters such as the initial concentration of metal ions, pH, adsorbent dosage and contact time. Some of the factors affecting the adsorption of heavy metal ions on the surface of GO include oxygenous functional groups in both the adsorbent and adsorbate, the thickness of the GO, the species of heavy metal ions in solution and the experimental conditions. It had been confirmed that the oxygenous functional groups on the GO surface played an important role in the adsorption [7].

Before the batch experiments, stock solutions of metal ions were prepared by dissolving certain amounts of PbCl_2_ for Pb^2+^, all in distilled water and then diluted to the desired initial concentration. The desired pH was adjusted by using an aqueous solution of 0.1 M HCl and 0.1 M NaOH. Throughout this exploration, flasks were shaken (agitated) at a constant rate, allowing sufficient time for adsorption equilibrium. It was assumed that the applied shaking speed allows all the reactive sites of the surface area of prepared materials to come into contact with heavy metal ions over the course of the shaking. The study was performed at room temperature to be representative of environmentally relevant conditions. All experiments were carried out in duplicate and the average value was used for further calculation.

The adsorbent was separated by filtration when the adsorption reached equilibration, and the concentrations of the residual Pb(II) ion were measured by an atomic absorption spectrophotometer (AAS).

To calculate for the adsorption capacities q_e_ (mg/g) the following Equation (1) was used:

(1) $$q_{e}=\frac{\left(C_{0}-C_{e}\right)\times V}{m}$$

where C_0_ and C_e_ refer to the initial and equilibrium concentrations of the metal ions (mg/L), respectively. V is the volume of the metal ion solution, and m is mass of the adsorbent.

The removal efficiency of the metal ions (%) was calculated using Equation (2):

(2) $$\mathrm{RE}\;\left(\%\right)=\frac{\left(C_{0}-C_{e}\right)}{C_{0}}\times 100\%$$

#### 2.6.1. Effect of Initial Metal Ions Concentration on the Adsorption

Using one set of sealed bottles; equal masses of 5 mg of prepared samples were added to 25 mL of Pb^2+^ solution with different initial concentrations such as 20, 40 and 60 mg/L respectively. Each bottle was agitated for 30 min at 200 rpm in a water bath shaker at 25 °C to allow equilibration. The supernatants were analysed as stated in the metal analysis after filtration of the adsorbent from the solution.

#### 2.6.2. Effect of pH on the Adsorption

The pH range of the experimental solutions of determined metal ions concentrations (stated above) was controlled at specifics of 3.5, 6 and 8.5 (a consistent difference of 2.5). This was ensured to establish a good check on the effect of pH in this experiment and secondarily for the purpose of keeping a check on the formation of metal hydroxides. 

#### 2.6.3. Effect of Contact Time on the Adsorption

Adsorbent masses, 5 mg each of Pure BC, BC/GO 1 and BC/GO 2 samples were added to 25 mL of Pb^2+^ solution with initial concentrations of 20, 40 and 60 mg/L. Each bottle was agitated for time intervals of 5, 10, 20, 30, 40, 50 and 60 min, respectively, at 200 rpm in a water bath shaker at 25 °C to allow equilibration. At the determined time, the residual concentrations were measured for the determination of adsorption capacities.

#### 2.6.4. Effect of Absorbent Dosage on the Adsorption

To further examine the prepared materials, the percentage of sequestration of Pb^2+^ was studied with three different specific mass dosages of the adsorbent; 5 mg, 10 mg and 15 mg (1/5 *w*/*v*, 1/2.5 *w*/*v* and 1/1.67 *w*/*v*, respectively).

#### 2.6.5. Adsorption Isotherms

A series of Pb(II) solutions with different initial concentrations were prepared by dissolving certain amounts of PbCl_2_ in deionized water. Quantities of 25 mL of the separately prepared solutions were experimented on by placing 5 mg of the adsorbent in each vial containing the solutions. Each vial was subjected to shaking for 2 h and then centrifugation followed that at 6000 rpm for 15 min and then, finally, the solutions were filtered. These solutions were then analysed by atomic absorption spectrophotometry (AAS) PG-990 (Beijing Purkinje General Instrument Co.,Ltd., Beijing, China). Blank solutions with the same concentrations as the adsorbents were subjected to the same procedure. Standard Pb(II) solutions were prepared in 0 (blank), 2, 4, 6, 8, 10 mg/L AAS stock solutions (Reagecon Diagnostics Ltd., Shanghai, China). Adsorption isotherms of the experiments were carried out at 25 °C and 40 °C. All samples and blanks were run in triplicate to ensure reproducibility and accuracy throughout the experimentation. 

Fitting the adsorption equilibrium data for Pb on adsorbents BC/GO 2 at temperatures of 25 °C and 40 °C with an initial concentration of 60 mg/L, the two main classical models applied in the solid–liquid adsorption systems, Langmuir and Freundlich equations were used. This was to help interpret the isotherm constants of the metal ions (Pb) binding to the adsorbent materials and their vital characteristics.

Langmuir’s model, as developed, poses to describe the adsorption on gas–solid phases and in a linear presentation given as Equation (3):

(3) $$q_{e}=\frac{Q_{\max}{\mathrm{bC}}_{e}}{{1+\mathrm{bC}}_{e}}$$

where q_e_ is the amount of metal ions adsorbed on the adsorbent at adsorption equilibrium (mg/g), Q_max_ (mg/g) is the maximum adsorption capacity, C_e_ is the equilibrium metal ion concentration in solution (mg/L) and b (L/mg) is the Langmuir binding constant related to the energy involved in adsorption. Plotting C_e_ versus C_e_/q_e_ gives a straight line with slope 1/Q_max_ and intercept 1/b Q_max_.

The Freundlich isotherm is commonly used for heterogeneous surface energy systems with a linear Equation presented in log form as Equation (4):

(4) $${\mathrm{logq}}_{e}={\mathrm{logK}}_{f}+\frac{{\mathrm{logC}}_{e}}{n}$$

where q_e_ (mg/g) is the equilibrium adsorption capacity, C_e_ (mg/L) is the metal ion equilibrium concentration in the aqueous solution, K_f_ is the Freundlich constant and n indicates the adsorption intensity in heterogeneous systems. K_f_ and n can be determined from the linear plot of log q_e_ versus log C_e_.

#### 2.6.6. Adsorption Kinetics Studies

A batch technique was used to analyse the adsorption kinetics for Pb^2+^ uptake on the prepared samples. Adsorbents (5 mg) were placed in each vial with 25 mL of PbCl_2_ (Pb) and conducted at 25 °C.

Initial concentrations of 20 mg/L, 40 mg/L and 60 mg/L of Pb(II) solutions were used. As time is of great importance in a kinetics experiment, the vials were agitated in a temperature-controlled bath shaker and then subsequently centrifuged at specified time (min) of 10, 20, 30, 40, 50 and 60. Concentrations of solutions were measured at the beginning and after the specified times, all determined by AAS. The quantities or amounts were calculated using the initial and final concentrations of Pb(II) in the aqueous phase. Again, all samples and blankets were run in triplicate to ensure accuracy and reproducibility. 

Adsorption kinetics are essential in the various approaches to design metal ions remediation systems, especially, in batch operation for large-scale processes [46]. There are two major kinetic models popularly applied in the study of adsorption, namely pseudo-first-order (PFO) and pseudo- second-order (PSO) models. We explored both models to find the best fit for the prepared materials in this experiment. Ideally, there are two key issues to address in the kinetic experimentation; contact time for equilibrium adsorption against the influence of the initial adsorbate concentration on the uptake.

The PFO equation developed by Largergen describes the rate of adsorption in the liquid-phase systems and is represented in a linear form in Equation (5):

(5) $$\frac{\mathrm{dqt}}{\mathrm{dt}}=k_{1}\left(q_{e}-q_{t}\right)$$

where q_e_ and q_t_ (mg/g) are the adsorption capacities at equilibrium (t_e_) and time t (min), respectively, and k_1_ (min^−1^) is the constant rate of the PFO kinetic model.

The PSO kinetic order model expression of Ho and Mckay [46] reveals a chemical bonding phenomenon with divalent metal ions and polar functional groups on surfaces which is assumed to cause cationic exchanges; in Ho and Mckay’s case on peat. The amount of adsorbed divalent ions (metals) on the surface of adsorbent at time (t) and equilibrium time (t_e_) is given in Equation (6):

(6) $$\frac{\mathrm{dqt}}{\mathrm{dt}}=k_{2}{\left(q_{e}-q_{t}\right)}^{2}$$

where k_2_ (g·min/mg) is the overall rate constant for the adsorption process, q_e_ (mg/g) is the amount of Pb(II) ions adsorbed at equilibrium and q_t_ (mg/g) represents the amount of Pb(II) ions adsorbed at any time as per experimentation. To aid in calculations, we considered boundary conditions for linearised form, the equation turns to become Equation (7):

(7) $$q_{t}=\frac{k_{2}q_{e}{}^{2}t}{1}+k_{2}q_{e}t$$

with $k_{2}q_{e}{}^{2}=h$, which defines the initial sorption rate. The initial sorption rate, h, the equilibrium sorption capacity, q_e_ and constant rate of PSO, K_2_ are derived from the slope and intercept of the plot t/q as against t.

#### 2.6.7. Desorption Experiments

To understand and determine the reusability of as-prepared samples, 50 mg of BC, BC/GO 1 and BC/GO 2 composites were weighed into 100 mL Erlenmeyer flasks, and 30 mL of Pb^2+^ metal ion solution 60 mg/L buffered at pH 8 was added. Aliquots were separated by filtration after subjecting them to agitation with the use of orbital shakers at 24 °C for 3 h at 120 rpm. Optimum adsorption parameters were ensured considering the functions of contact time, pH (initial) of solution and the initial metal ions’ concentration. After filtering, they were washed with deionized water and the adsorption capacities were ascertained. After a while, the samples loaded with Pb^2+^ were weighed into a 100 mL Erlenmeyer flask and 20 mL of 0.1 M HNO_3_ and 0.1 M HCl were added and stirred for 5 min (130 rpm) at 24 °C. The final concentrations were detected with AAS. The determination of the desorption ratio was derived from the amount of metal ions adsorbed on adsorbent materials and the final PbCl_2_ ions concentration in the adsorption medium. The desorption ratio was calculated from the following Equation (8):

(8) $$\mathrm{desorption}\;\mathrm{ratio}\;\left(\%\right)=\frac{{\mathrm{amount}\;\mathrm{of}\;\mathrm{Pb}}^{2+}\;\mathrm{desorbed}}{{\mathrm{amount}\;\mathrm{of}\;\mathrm{Pb}}^{2+}\;\mathrm{adsorbed}}\times 100\%$$

## 3. Results and Discussion

### 3.1. Characterisation of Adsorbents

#### 3.1.1. FTIR Analysis

**Figure 2:**
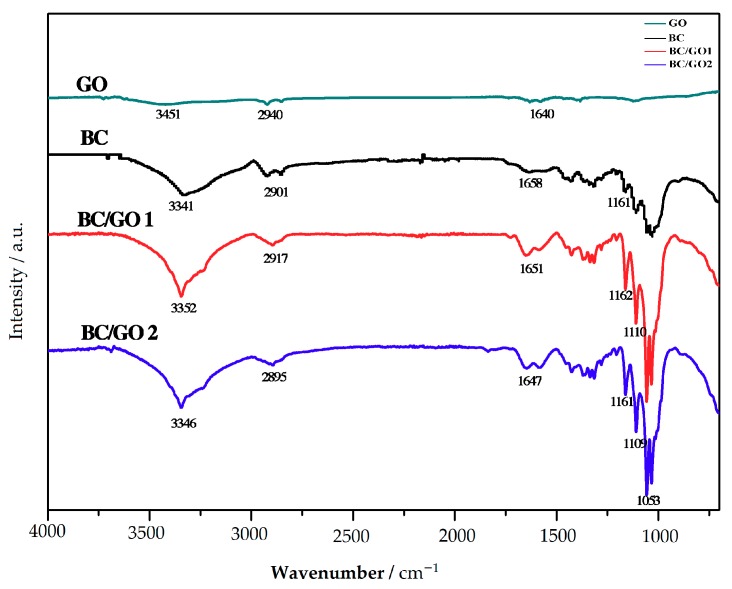
FTIR spectra of GO, BC, BC/GO 1 and BC/GO 2.

For Pure BC, peaks of 3341 cm^−1^ represent a hydrogen bond, and, as could be seen from Figure 2, sharp peaks of 3352 cm^−1^ and 3346 cm^−1^ for BC/GO 1 and BC/GO 2, respectively, are all representations of the (O-H stretching) of hydrogen bonding. These peaks confirm the existence of hydroxyl groups in the samples prepared, which are essential to the adsorption efficiency. The sharp adsorption peaks at 2901 cm^−1^, 2917 cm^−1^ and 2895 cm^−1^ of pure BC, BC/GO 1 and BC/GO 2, respectively, represent (i) asymmetric CH_2_ stretching for pure BC and BCGO 1, and (ii) Symmetric CH2 stretching for BC/GO 2. The C-H stretching vibration detected proves the existence of alkyl groups in the composite material. Peaks at 1651 cm^−1^ and 1647 cm^−1^ for both BC/GO 1 and BC/GO 2, respectively, show Amide 1 (C=O) stretching; confirming the existence of the ester group which corresponds to the stretching of the carbonyl of carboxylic acid groups. This is due to the surface interaction between GO and BC. Peaks at 1161 cm^−1^ and 1053 cm^−1^ are attributed to the increase in ether bond length. In summary, there was successful esterification between BC and GO. The confirmation of the existence of hydroxyl groups, alkyl groups and carboxylate groups suggests the prepared materials have a high capacity to enhance the adsorption of metal ions.

#### 3.1.2. TGA Analysis

**Figure 3:**
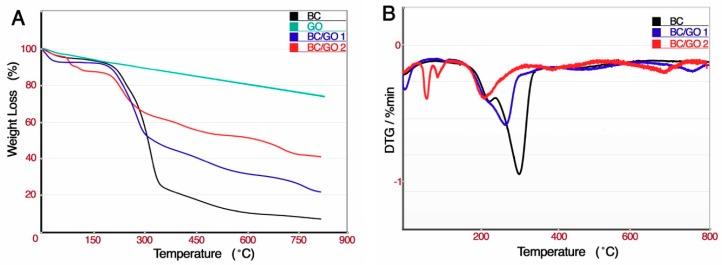
TGA analysis of prepared samples.

The thermal stabilities of the BC/GO nanomaterials were evaluated using thermogravimetric analysis (TGA). Almost all sample curves show their first mass loss from room temperature to 100 °C, confirming the presence of water. As shown in Figure 3A, GO shows a continuous steady weight loss of about 28.8% when heated to 800 °C, this could be attributed to some organic group defects on the surface of the GO sheets compared to BC which shows intermittent irregular weight loss; first slightly at a temperature <50 °C. Then, a slow loss to <250 °C (at around 89%) before a further large weight loss (about 74%) to 360 °C, and a final 14% weight loss at 800 °C as a result of depolymerisation and the decomposition of the cellulose backbone. Both BC/GO 2 and BC/GO 1 composites experienced a couple of additional weight degradation steps during the thermal process with a mass retention of 42% at 800 °C; the presence of a good amount of GO may contribute to this retention capability. BC/GO 1 compared to BC/GO 2 shows slightly fewer steps in degradation with the percentage of retention seemingly lower than BC/GO 2 at 800 °C, which was at 24%.

Results revealed that GO remarkably improves the thermal stability of BC/GO composites, BC/GO 2 was discovered to contain about 15% more GO than BC/GO 1 from the TGA information. However, conclusively, the prepared BC/GO composites can be said to have a good to decent level of mass retention at higher temperatures.

#### 3.1.3. XRD Analysis

**Figure 4:**
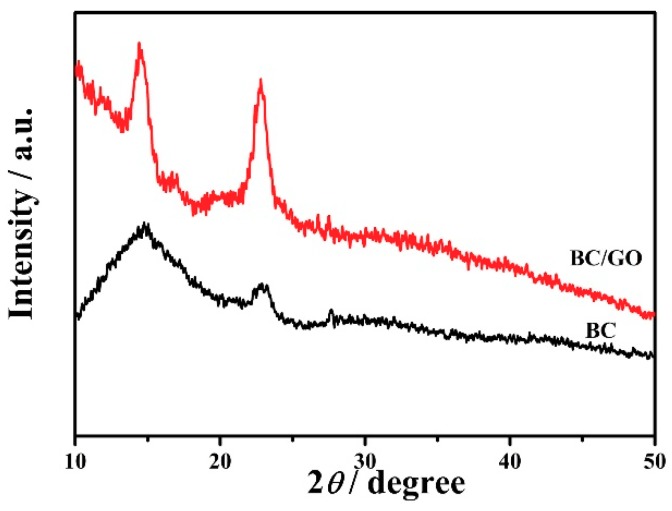
X-ray diffraction patterns of pure BC and BCGO.

The crystalline structure of the prepared samples was characterised by X-ray diffractograms of cellulose, presented in Figure 4. Typical peaks are easily observable in sample patterns of BC; 2θ = 14.2°, 23.1° and a small peak at 28°, which were consistent with diffraction (110) and (200) planes of cellulose I. BC/GO exhibits similar diffraction peaks at 2θ = 14.1°, 22.8° and a small peak at 28° at a somehow higher intensity with no significant peaks shifting or no appearance of new peaks. The higher intensity can be attributed to the introduction of GO onto the surface of BC as they (GO) are evenly dispersed on the surface inside the BC matrix. This also confirms the physicochemical modification of the BC, helping to enhance the degree of crystallinity of the BC to about 20–30%. The chemical modification purportedly occurs at the surface of the cellulose fibres [47]. Further data information is offered to support the nature of the thermal stability of the materials (BC/GO 1 and BC/GO 2) divulged by the TGA results in Figure 3a,b.

#### 3.1.4. SEM Analysis

**Figure 5:**
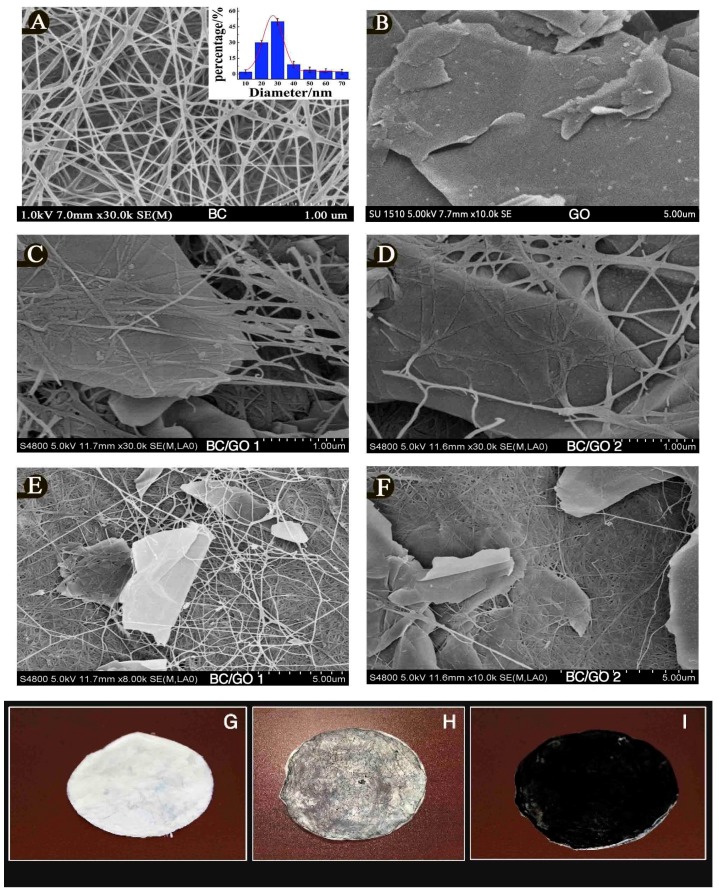
SEM images of samples (**A**–**F**) at 200x and 10,000x magnification. (**G**–**I**) Photographed images of final samples; (**G**) pure BC (**H**) BC/GO 1 and (**I**) BC/GO 2.

SEM images of pristine BC, BC/GO 1 and BC/GO 2 samples are displayed in Figure 5A–F, with BC showing a nanoporous three-dimensional morphological network structure. The microfibrils are randomly arranged by overlapping and intertwisted cellulose ribbons in irregular planes across the span of the membrane with nanosized empty spaces between them [21]. BC/GO 1 and BC/GO 2 provides a good understanding of how GO is introduced into the matrix of BC during synthesis. Typical BC features the archetypal three-dimensional nanosized spaces between the filaments, which is further elucidated by the inserted graph on Figure 5A. The filament size is between 10 to 70 nm according to the insert on Figure 5A. Figure 5B shows GO sheets that are well exfoliated along the planes and evenly distributed across the entire membrane area, appearing clean and smooth. Figure 5C,E represent BC nanofibrils successfully cross-linked with GO sheets in a manner physically seen as entanglement but also chemically explained by the covalent bonding interaction between the reactive groups and the intercalation of GO sheets with BC fibers; typically, the images representing BC/GO 1. The same can be described for BC/GO 2, see Figure 5D,F; however, it contains more GO than BC/GO 1. GO sheets are clearly interlocked within the 3D web-like arrangement of the BC nanofibrils; which occurs during the formation of the structure by the in situ cultivation approach. BC/GO 1 and BC/GO 2 images depict similar SEM images and can be explained by the same mode of composition; chemically, as covalent bonding occurs between the reactive groups of both BC and GO. However, a slight difference can be seen from the photographed images in Figure 5G–I with regard to the appearance of both materials. As purposely engineered during the entanglement process in the culture medium, a greater amount of GO is evidently trapped on the surface of the liquid medium face beneath the BC of BC/GO 2. This explains the coin-effect (plain on one side/black (GO) on the other side).

The SEM images, Figure 5C–F, show well-composited materials with micro-spaces for ion transport. Conclusively, a strong adhesion between GO and BC by way of entanglement and strong electrostatic (physicochemical) interactions between the OH groups of BC and GO confirm successfully biosynthesized membrane materials BC/GO 1 and BC/GO 2. 

#### 3.1.5. Analysis of Specific Surface Area (SSA) and Pore Size Distribution (PSD) of BC/GO Adsorbent

**Figure 6:**
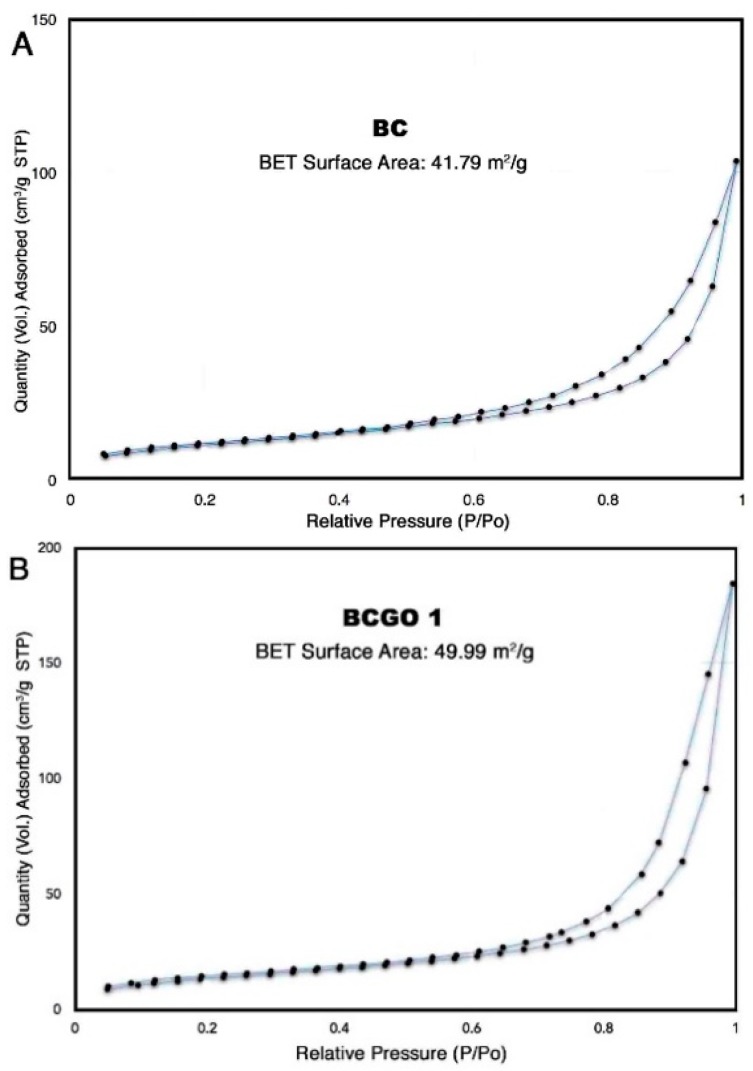

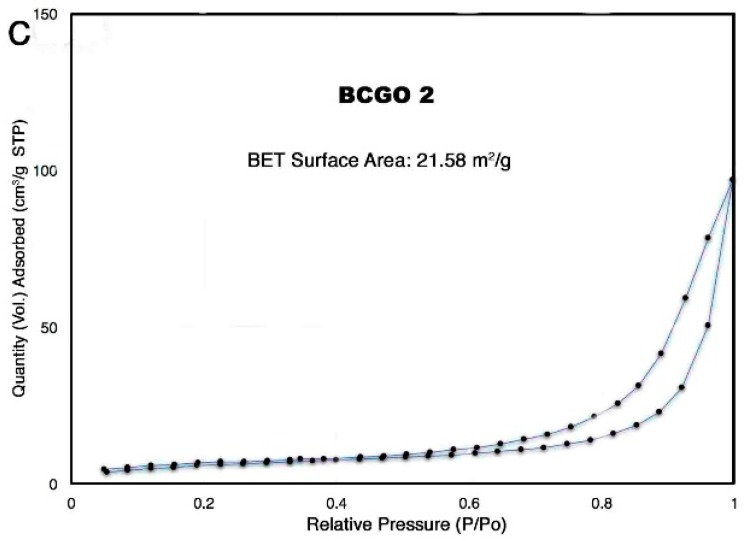
Nitrogen adsorption/desorption (at 77 K) curves (specific surface area) distribution curves of (**A**) BC, (**B**) BC/GO 1 and (**C**) BC/GO 2.

Samples of BC, BC/GO 1 and BC/GO 2 tested for their textural properties according to the BET (Brunauer, Emmett and Teller) and BJH (Barrett, Joyner and Halenda) approach calculations are presented as follows: BC/GO 1 has a specific surface area (BET) of 49.99 m^2^/g as seen in Figure 6B, while displaying an average pore size of 18.696 Å with a total pore volume of 0.0356 cm^3^/g. Nitrogen sorption experiments at 77 K of BCGO 2, see Figure 6C, displays a specific surface area (BET) of 21.58 m^2^/g with an average pore size of 25.388 Å and a total pore volume of 0.0307 cm^3^/g. Pakulski et al. report the GO specific surface area to be 10 m^2^/g and confirmed that GO falls within the range of 10 to 100 m^2^/g depending on the inherent water content level by BET calculation [48]. In comparison to GO, BC samples are reported to have a SSA of 77 m^2^/g by Pircher et al., which is close to 41.79 m^2^/g, see Figure 6A, as displayed per this experiment’s sample BC [49] with an average pore size of 17.111 Å. Both BC/GO 1 and 2 are categorised as microporous or mesoporous materials under the pore size specification of the International Union of Pure and Applied Chemistry (IUPAC) which classifies pores under 20 Å as microporous, pores between 20 Å to 500 Å as mesoporous and pores over 500 Å as macroporous materials. The mesoporous nature of our BC/GO composites sets to enhance the uptake and facilitate ion transport kinetics in the chelation (electrostatic interactions) of heavy metal ions [50]. The specific surface area of BC/GO 2 serves as a great contributor to the high adsorption capacity recorded in this experiment.

### 3.2. Adsorption Experiments

#### 3.2.1. Effect of Initial Metal Ions Concentration on Metal Ion Removal

**Figure 7:**
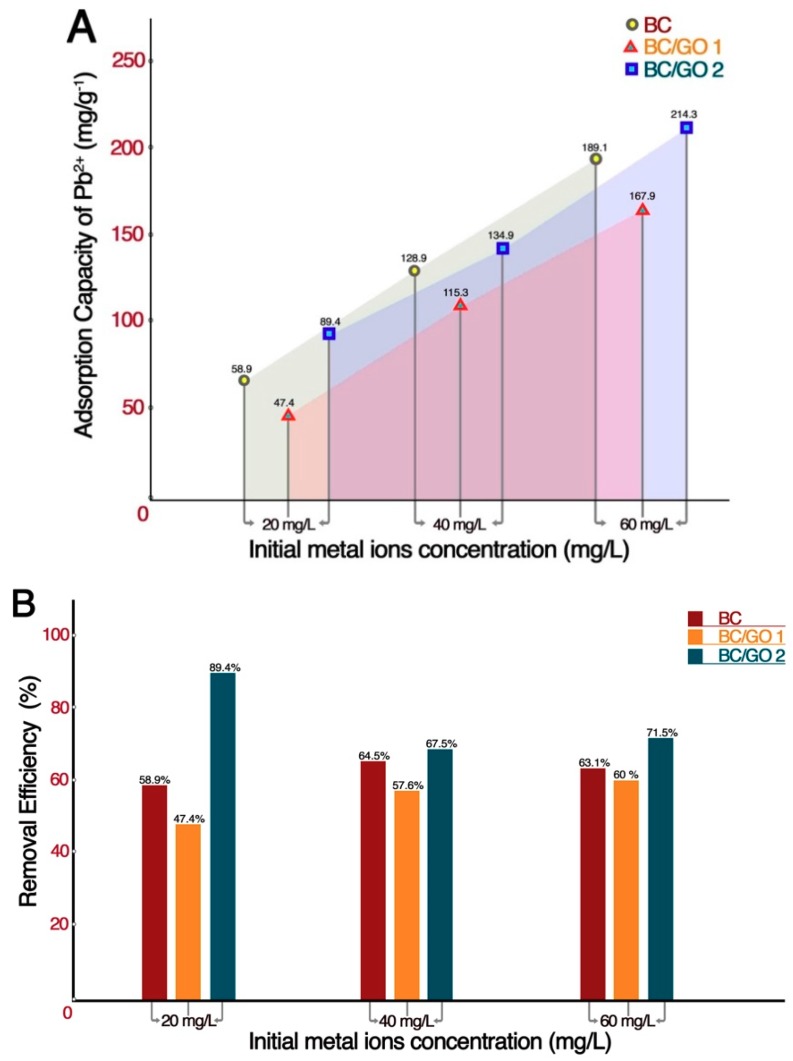
Effect of the initial concentrations of the metal ions upon ion removal. (**A**) Adsorption capacity in mg/g. (**B**) Removal efficiency in percentage %.

Three distinctive initial ions concentrations were experimented in this study: 20, 40 and 60 mg/L. It can be seen from Figure 7A that the adsorption capacity of Pb(II) ions increases with an increase in initial metal ions’ concentration values in the medium. The maximum adsorption is seen to be 214.3 mg/g for the 60mg/L initial concentration sample solution onto the BC/GO 2 adsorbent material. BC and BC/GO 1 recorded 189.1 and 167.9 mg/g, respectively, for the 60 mg/L initial concentrations solution medium. Evidently, BC/GO 2 always records the highest capacity followed by BC with BCGO 1 demonstrating the least capacity adsorption in all the solution categories. This suggests an interesting discovery since pure BC is considered theoretically to require modification to increase its adsorption capacity; hence the composition with GO for modification in this study. BC/GO 2 proved to have a notable enhancement in the adsorption efficiency whereas BC/GO 1, with the successful bonding (50% less GO content than BC/GO 2 according to Table 1) with BC, had lower capacities than pure BC. This may be due to a phenomenon we intend to investigate in our follow-up publication on the same subject. The higher adsorption capacity of BC/GO 2 is due to the abundant functional groups on the material serving as adsorptive sites for interaction with metal ions (Pb^2+^) for chelation. SEM and photographed images plus FTIR results confirm this. The highest removal efficiency (%) derived in this experiment is 89.4%, as can be seen in Figure 7B. 

#### 3.2.2. Effect of the Solution pH on Metal Ion Removal

**Figure 8:**
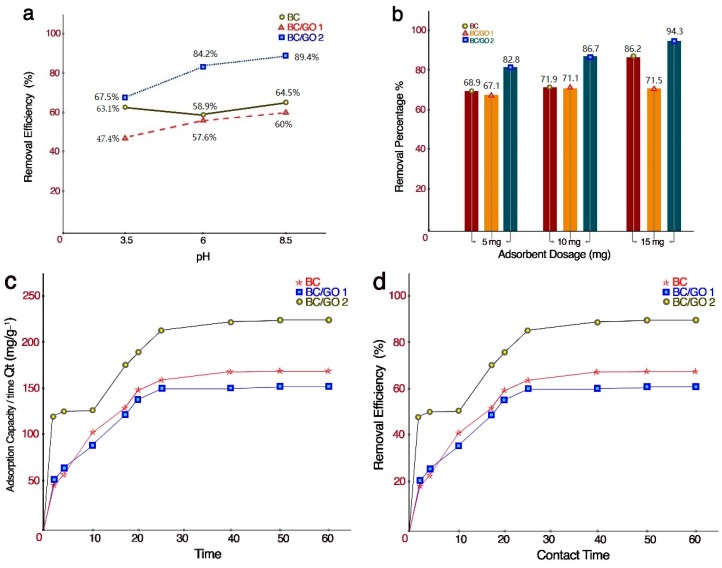
(**a**) Effect of solution pH on removal (**b**) Effect of adsorbent dosage on metal ion removal (**c**,**d**) Effect of contact time on metal ion removal.

Figure 8a reveals the effect of the three different initial concentrations at 25 °C at an equilibrium time t_e_ (min) of 1 h set at pH 3.5, 6 and 8.5; showing the highest removal efficiency of 89.4% at pH 8.5 with the BC/GO 2 material. This proves that the prepared materials perform best in higher pH conditions for Pb^2+^ adsorption. Several publications [27,51,52,53] report that Pb(II) ions removal capacity increases with a pH increase but the highest pH value recorded by most of these publications is ≤6. Its predominantly reported that at lower pH values, Pb(II) tends to be difficult to remove as a repulsive force between the metal cations and most of the functional groups on cellulose occurs due to protonation.

At pH 6, the adsorption capacity (in this experiment) is 84.2% (which is slightly below the highest value (8.5)) overall, until it plateaued. These high values agree with most reports which have found that the pH range within which Pb(II) ions are most greatly absorbed is between 4.5 to 6. However, it is also widely established that the precipitation of metal ions occurs at higher pH values over 8–11. In this study, minimal to almost no precipitation was witnessed even at pH 8.5 when the recorded percentage of the removal efficiency was 90%. The high adsorption of Pb(II) onto BC/GO 2 is attributed to the availability of numerous hydroxyl and carboxyl groups on the material which are ionised under high pH (but not beyond 8) conditions to enhance the strong electrostatic attraction with metal ions in the solution (Pb^2+^). Therefore, the pH value of most solutions was set at 6 for the following experiments due to its positive effect.

#### 3.2.3. Effect of Contact Time on Metal Ion Removal

Figure 8c,d show a sharp adsorption or uptake within the first 5 min for all the materials with BC/GO 2 expressing high values with time compared to BC, with BC/GO 1 showing the lowest adsorption capacity of the three. Similar uptake speeds can be identified for all samples as adsorption is sharp for the first 5 min and steadily rises from 10 min and begins to experience equilibrium from 28–30 min. The reason for the sharp uptake within 5 min could be due to the free surface area of the materials available for bonding with the metal ions. However, as time increases and surface reactive areas are sufficiently occupied through bonding exhaustion, the uptake plateaus, showing that it has reached equilibrium. An uptake equilibrium in all the material adsorption cases begins at 28 min and is maintained as time progresses.

#### 3.2.4. Effect of Adsorbent Dosage on Metal Ion Removal

The result of the adsorbent dosage is displayed in Figure 8b where a trend is evident as the removal efficiency increased with an increase in the adsorbent dosage. It is seen that BC/GO 2, with the highest adsorption efficiency, registers removal efficiencies of 82.8%, 86.7% and 94.3% for a dosage weight of 5, 10 and 15 mg, respectively. BC/GO 1 and pure BC follows the trend. The increase in efficiency is explained by an increase in the adsorptive reactive sites and surface area in the aqueous system for greater sequestration.

### 3.3. Adsorption Kinetics

**Figure 9:**
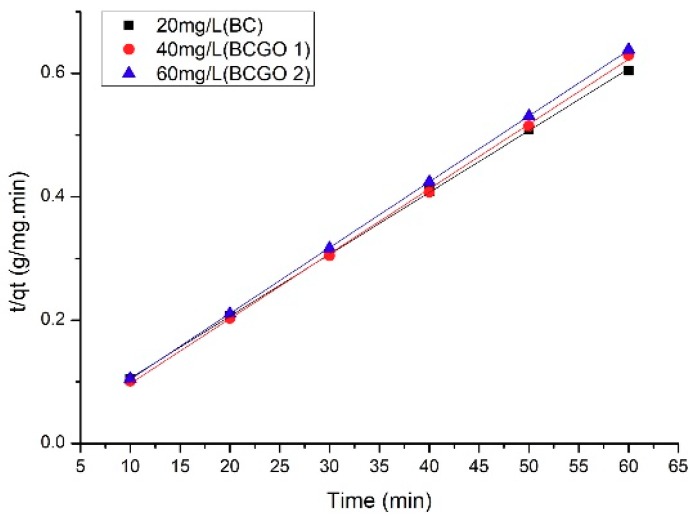
Pseudo-second-order (PSO) rate model plot of Pb(II) adsorption on to prepared samples.

Figure 9 depicts the corresponding PSO for Pb(II) ion adsorption on to BC, BC/GO 1 and BC/GO 2 because it agrees with the linear regression plotting chosen for this experiment whereas PFO did not conform. Both PFO and PSO were used to model the adsorption rate of metal ions onto the surface of prepared membranes at pH 6–8 (experimental optimum) at 25 °C and 130 rpm. Using origin Pro 2016 software, the kinetic data, shown in Table 2, was modelled by linear regression analysis using a PFO and PSO equation model. It was discovered that the adsorption data of Pb^2+^ on BC, BC/GO 1 and BC/GO 2 corresponded seamlessly with PSO, see Figure 9, and the plots show a high correlation coefficient (R^2^), complying well with trusted theoretical assumptions of the PSO.

Parameters derived from the plots for Pb(II) adsorption are shown in Table 2.

The data, shown in Table 2, further indicate that the adsorption is predominantly controlled by chemisorption which is interpreted as a surface complexation of metal ions onto the abundant oxygenous groups found on the reactive sites on the surface of GO as also asserted by [54]. The data also reveal the observed increase in the uptake capacity was respondent to higher initial concentrations of metal ions (Pb^2+^) solution in shorter times.

### 3.4. Adsorption Isotherms

**Figure 10:**
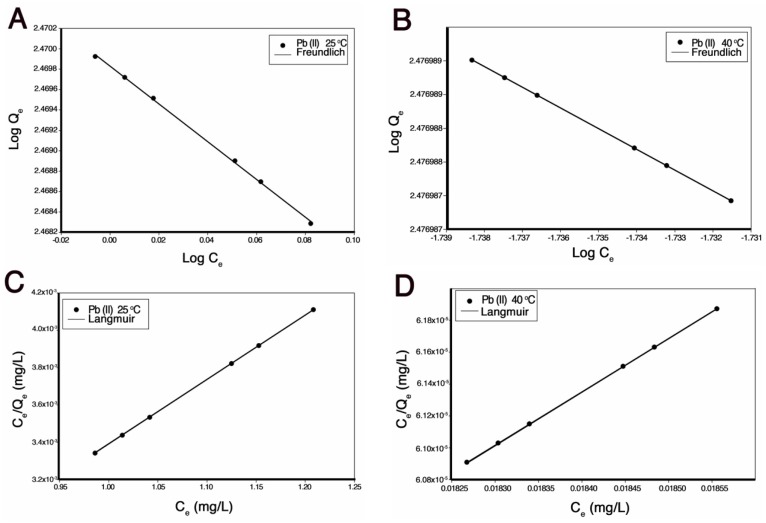
Adsorption isotherms of Pb(II) ions on BC/GO 2 at temperatures of 25 °C and 40 °C pH = 6. (**A**) Freundlich model Plot 25 °C, (**B**) Freundlich model Plot 40 °C, (**C**) Langmuir model Plot 25 °C, (**D**) Langmuir model Plot 40 °C.

From experimental equilibrium data of the Langmuir (Q_max_) and the Freundlich models (Q_max_), the maximum metal ion adsorption capacity of the adsorbent exhibited the following order: Pb^2+^ 40 °C-Langmuir > Pb^2+^ 25 °C-Langmuir, whereas Pb^2+^ 40 °C-Freundlich > Pb^2+^ 25 °C-Freundlich; which could be seen from plots in Figure 10, suggesting that the adsorbent is more of a monolayer homogeneous adsorption material rather than being heterogeneous.

Judging from the obtained coefficients given in Table 3, it can be concluded that the Langmuir equation shows a better fit (R^2^ = 0.99), with a higher Q_max_ (mg/g) maximum adsorption capacity to the experimental data than the Freundlich equation. The highest maximum adsorption capacity Q_max_ (mg/g) was 303.0 mg/g.

### 3.5. Desorption and Re-Adsorption

The samples loaded with Pb^2+^ were weighed into a 100 mL Erlenmeyer flask and 20 mL of 0.1 M HNO_3_ and 0.1 M HCl were added and stirred for 5 min (130 rpm) 24 °C. The final concentrations were detected with AAS.

**Figure 11:**
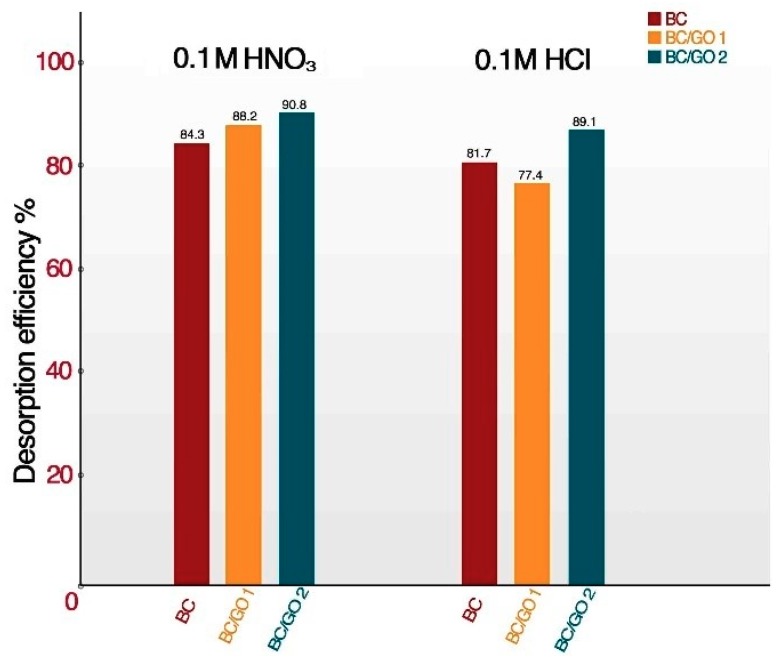
Desorption results with the use of HNO_3_ and HCl for Pb^2+^.

There were three cycles of adsorption­–desorption–re-adsorption process with the average taken to be representative of the desorption and re-adsorption efficiency of the adsorbents as a percentage (%). From the results in Figure 11, it is easy to indicate that HNO_3_ performed slightly better than HCl by a minimal margin. The highest desorption percentage for HNO_3_ is 90.8% and 89.1% for HCl. 

Performing re-adsorption using the BC, BC/GO 1 and BC/GO 2, which were desorbed using HNO_3_, adsorbents demonstrated efficiencies for Pb^2+^ close to 95%. These results suggest that both prepared adsorbents and adsorbate (Pb^2+^) can be recovered and reused for about three times before there will be a significant loss of the adsorption capacity. 

These prepared materials, especially BC/GO 2, represent efficient, cost-effective and potential adsorbents for metal ions removal due to their excellent reusability performance.

## 4. Conclusions

This work successfully prepared a green bacterial cellulose graphene oxide composite material; physicochemical modification (confirmed by XRD) by means of esterification between BC and GO (confirmed by SEM) with good levels of mass retention at higher temperatures (TGA) and found by FTIR to richly contain hydroxyl groups, alkyl groups and carboxylate. Adsorption studies revealed the material to have a very high removal efficiency of over 90% at pH 6–8.5 with increasing adsorption capacity as parameters such as initial ions concentration, adsorbent dosage and contact time increase. Adsorption data corresponded with trusted theoretical assumptions of the PSO indicating predominant chemisorption-controlled adsorption interpreted as a surface complexation of metal ions onto the abundant oxygenous groups found on prepared materials. Isotherm results had greater affinity with Langmuir showing the highest maximum adsorption capacity (Q_max_) 303.03 mg/g than Freundlich; hence Langmuir’s model best describes the adsorption system. Finally, desorption and re-adsorption experiments show that both the adsorbent and adsorbates can be over 90% desorbed using HNO_3_ or HCl and reused at an efficiency of 95%. An efficient and potentially green adsorbent for metal ions removal, especially Pb^2+^, from an aqueous system was produced. Compared to other adsorbents, BC/GO 2 outperforms others recently reported for the removal of Pb^2+^, as shown in Table 4.

Follow up research is necessary to optimize the adsorption capability of BC/GO 1, the handleability of the best adsorbent material (BC/GO 2), reusability of both materials, as well as to check the feasibility with real industrial effluents or groundwater sample removal with Pb(II) present.

## Figures and Tables

**Table 1:** Sample code, GO-content, percentage of GO trapped and cultivation period.

Code (Name)	GO-Content (% *w*/*v*) into Medium	GO% Trapped	Cultivation Period
BC	0	0	20 days
BC/GO 1	0.1	40%	20 days
BC/GO 2	0.1	95%	20 days

**Table 2:** Results of experimental kinetic data for adsorption of Pb^2+^ onto BCGO materials.

Metal Ion/Material	q_e_ ex (mg/g)	Pseudo-First-Order (PFO) Model			Pseudo-Second-Order (PSO) Model		
		K_1_ (min)	q_e_ (mg/g)	R^2^	K_2_ (min·g/mg)	q_e_ (mg/g)	R^2^
BC	68.8	0.71	67.1	0.1821	1.75	68.9	0.998
BCGO 1	149.5	0.73	144.7	0.2343	1.75	149.8	0.998
BCGO 2	220.9	0.73	220.6	0.2976	1.75	221.8	0.999

**Table 3:** Isotherm parameters for the adsorption of Pb^2+^ onto BC/GO materials.

Isotherm Model	Temperature	R^2^ Values	Q_max_ (mg/g)	b (L/mg)	n	Kf
Langmuir	25 °C	0.99	285.7	1.06	-	-
	40 °C	0.99	303.0	1.75	-	-
Freundlich	25 °C	0.98	294.2	-	1.67	2.07
	40 °C	0.98	299.9	-	1.47	2.01

**Table 4:** This paper’s results when comparing different adsorbent materials reported recently in the literature.

Sorbent Material	Adsorbate/Metal Ion	High Adsorption Capacity (mg/g)	Ref.
Chitosan modified by graphene oxide	Cu(II)	202.5	[55]
Cross-linked graphene oxide sheets via modified extracted cellulose	Cu(II) and Pb(II)	46.39 and 186.48	[29]
Polyethylenimine coated bacterial cellulose	Cu(II) and Pb(II)	61.46 and 116.41	[56]
Chemically modified cellulose (DTD)	Cu(II) and Pb(II)	157.3 and 153.5	[57]
Modified gum tragacanth/graphene oxide composite	Pb(II), Cd(II), and g(I)	142.50, 112.50 and 132.12	[37]
Few-layered graphene oxide nanosheets (FGO)	Pb(II)	842/400	[31]
Carboxymethylated-bacterial cellulose CM-BC	Cu(II) and Pb(II)	12.63 and 60.42	[53]
GO/cellulose membranes	Pb(II)	107.9	[54]
This paper	Pb(II)	303.0 mg/g	
